# Whitening Effects of a Novel Oral Care Gel with Biomimetic Hydroxyapatite: A 4-Week Observational Pilot Study

**DOI:** 10.3390/biomimetics5040065

**Published:** 2020-11-24

**Authors:** Sonja Steinert, Joern Kuchenbecker, Frederic Meyer, Barbara Simader, Kai Zwanzig, Joachim Enax

**Affiliations:** 1 Dental Practice, Mauerstraße 8, 33602 Bielefeld, Germany; heckel.sonja@gmx.net (S.S.); info@praxis-zwanzig.de (K.Z.); 2 Dental Practice, Meinigstraße 29 A, 38667 Bad Harzburg, Germany; joernkuchenbecker@hotmail.com; 3 Dr. Kurt Wolff GmbH & Co. KG, Research Department, Johanneswerkstr 34–36, 33611 Bielefeld, Germany; barbara.simader@drwolffgroup.com

**Keywords:** teeth, biomimetic hydroxyapatite, oral care, tooth whitening, dentin hypersensitivity, observational study

## Abstract

The whitening effects of an oral care gel based on particulate microcrystalline hydroxyapatite, Ca_5_(PO_4_)_3_(OH), were tested in a 4-week observational pilot study. Patients were recruited from two dental practices in Germany. Participants were asked to complete a questionnaire regarding their personal perception of their tooth color and brightness as well as the level of dentin hypersensitivity at the baseline and after 4 weeks of twice daily use of an oral care gel with hydroxyapatite. Data of 25 patients with a mean age of 46 ± 16 years were analyzed. Various subjective whitening parameters showed a tendency to be improved after the 4-week use. Additionally, patients reported that symptoms of dentin hypersensitivity were significantly reduced (*p* < 0.05, 95% confidence interval (CI): (0.8; 2.4)), and the tooth surface was significantly smoother (*p* < 0.05, 95% CI: (0.54; 1.6)). In conclusion, microcrystalline hydroxyapatite is a promising whitening agent for oral care formulations and represents a biomimetic alternative to other whitening agents for daily dental care.

## 1. Introduction

Particulate hydroxyapatite, Ca_5_(PO_4_)_3_(OH), is a multifunctional biomimetic active ingredient used in preventive oral health care [1,2,3,4,5,6], and has a wide range of applications in this field. Specifically, it has been shown to remineralize both enamel and dentin, prevent caries [2,5,7,8,9,10,11,12], reduce bacterial colonization on teeth and dental surfaces [1,13], improve periodontal health [1,4,13,14,15], and improve symptoms associated with dentin hypersensitivity [3,16,17]. An important advantage of hydroxyapatite compared to other active ingredients in preventive oral health care is its high biocompatibility as it has no side effects when used in daily oral care [6,18,19,20,21].

Tooth whitening continues to be one of the most requested elective dental procedures by the public [22]. Home use whitening-products become more and more popular, even in younger age-groups. However, tooth whitening procedures are not recommended for those under the age of 18, as side effects may occur [23]. However, hydroxyapatite-based oral care products are well-suited for all age groups. Several in vitro and in situ studies have analyzed the interaction of biomimetic hydroxyapatite particles with enamel surfaces [1,24,25,26]. This interaction can lead to a tooth whitening effect [6], which has been shown in different in vitro [27,28,29] and in vivo studies [17,30,31]. Unlike peroxide-based products which may induce side effects (e.g., bleaching sensitivity and damage of the organic matrix of enamel and dentin), hydroxyapatite can be used on a daily basis without any adverse reactions, and even from persons under the age of 18 [22]. Due to the high chemical, structural, and mechanical similarity of biomimetic hydroxyapatite to human enamel crystallites [24], hydroxyapatite offers gentle tooth cleaning properties without being abrasive; which is an important advantage over other whitening agents like alumina and perlite [22,32].

Hydroxyapatite, the active ingredient, can be used, for example, in toothpastes [2,3,4,8] and mouthwashes [1,24]. Additionally, hydroxyapatite is commercially available at higher concentrations in gels to enhance the effects of daily tooth brushing [9,11,29,33]. Recent in vitro studies show that a newly developed hydroxyapatite gel has significant whitening [29] and enamel remineralizing effects [9] as well as erosion protective properties [33]. Amaechi et al., for example, showed that this hydroxyapatite-gel is as effective as a highly concentrated fluoride gel with 12,500 ppm fluoride regarding remineralization of early caries lesions [9]. The hydroxyapatite gel can be used daily, whereas the application of highly concentrated fluoride gels is limited to once a week [9].

The aim of this observational pilot study is to analyze the general suitability of this novel hydroxyapatite-based oral gel in daily dental care as well as to analyze its tooth whitening effects.

## 2. Materials and Methods

### 2.1. Recruitment, Inclusion and Exclusion Criteria

This observational study was performed as an open-label, uncontrolled, pre-post interventional study design. Patients were recruited at two dental practices in Germany (Bielefeld and Bad Harzburg). The following inclusion and exclusion criteria were verified at the dental practices:

Inclusion criteria:

- Age ≥ 18 years
- Written informed consent
- Overall good oral health status
- Maximum of one restoration on vestibular surfaces of teeth 13–23 and 33–43

Exclusion criteria:

- Severe periodontitis
- Severe dental erosion
- Untreated carious lesion(s)
- Bleaching with peroxides (in-office or at home) 4 weeks prior to their participation in this observational study

Additionally, patients were asked not to use peroxide-based oral care products (in-office and at home) during the 4-week study period. Patients were informed about the background and the aim of the study at the dental practices and signed an informed consent prior to their participation in this observational study. The safety of the hydroxyapatite oral gel was confirmed by a safety assessment according to the EU-regulations on cosmetic products [34].

### 2.2. Composition of the Hydroxyapatite Gel

The whitening effects of a commercially available oral gel in original packaging with the active ingredient microcrystalline hydroxyapatite were analyzed (Karex gelée, Dr. Kurt Wolff GmbH & Co. KG, Bielefeld, Germany; cosmetic product). The hydroxyapatite used in Karex gelée was thoroughly characterized by physicochemical methods such as X-ray powder diffraction (XRD) and scanning electron microscopy (SEM). It shows a high similarity to natural human enamel crystallites both chemically and structurally [24,35]. Moreover, this hydroxyapatite’s ability to remineralize enamel has been studied under in situ conditions [8].

Karex gelée contains the following ingredients (according to the International Nomenclature of Cosmetic Ingredients):

Aqua, Hydroxyapatite, Glycerin, Hydrogenated Starch Hydrolysate, Calcium Lactate, Hydroxyethylcellulose, PEG-40, Hydrogenated Castor Oil, Xylitol, Calcium Carbonate, Hydroxyacetophenone, 1,2-Hexanediol, Caprylyl Glycol, Aroma, Stevia Rebaudiana Leaf/Stem Powder, Propylene Glycol, Sodium Hydroxide, Limonene, and Citral.

### 2.3. Application of the Hydroxyapatite Gel

The hydroxyapatite gel was applied in the morning and in the evening directly after tooth brushing by finger (1 cm gel for teeth of the upper jaw, and 1 cm gel for the lower jaw) for 28 days. The patients were asked not to rinse out their mouth with tap water after the application of the gel. Patients were instructed to continue their regular oral hygiene regimen with respect to brushing and flossing.

### 2.4. Questionnaires

The following questions were answered by the patients at baseline and after 28 days (follow-up). Patients were asked to answer highly subjective questions by using the visual analogue scale (VAS) [17,36]. Note that the VAS did not contain any measuring units visible for the patients.

(A) Baseline questionnaire
  General questions:
  (1) Age
  (2) Gender
  (3) Currently used toothpaste
  (4) Currently used toothbrush (options: electric toothbrush, manual toothbrush, or sonic/ultrasonic toothbrush)
  (5) Which products do you use for tooth whitening at home? (options: no whitening product, whitening toothpaste, whitening mouthwash, whitening stripes, or other whitening products)
  (6) How often do you use products for tooth whitening at home? (options: never, less than once a month, 1–3× per month, 1–3× per week, or 4–6× per week, daily)
  (7) How often were your teeth bleached at a dental practice in the entire year 2019? (options: never, 1× per year, 2× per year, or more than 2× per year),
  (8) Did you feel any unwanted effects after in-office bleaching? (options: no bleaching was performed, I did not feel any unwanted effects, sensitive teeth, or gum problems)
  Questions related to tooth color:
  (9) How do you describe the color of your teeth? → VAS: yellowish/grey (0.0 cm), white (10.0 cm)
  (10) How do you describe the brightness of your teeth? → VAS: dark (0.0 cm), bright (10.0 cm)
  (11) How satisfied are you with the color of your teeth? → VAS: very dissatisfied (0.0 cm), satisfied (10.0 cm).
  (12) How do you think other people assess your teeth/tooth color? → VAS: unattractive (0.0 cm), attractive (10.0 cm).
  Questions related to tooth sensitivity, tooth surface, and mouthfeel:
  (13) How sensitive are your teeth (cold beverages/food, ice, cold air, etc.)? → VAS: very high tooth sensitivity (0.0 cm), no tooth sensitivity (10.0 cm)
  (14) How do you assess the surface of your teeth (by using the tongue)? → VAS: rough (0.0 cm), smooth (10.0 cm).
  (15) How does your mouth feel? → VAS: unpleasant (0.0 cm), pleasant (10.0 cm)
(B) Follow-up questionnaire
  For follow-up only questions No. 9–15 were asked again; Furthermore, patients could add additional comments (No. 16):
  Questions related to tooth color
  (9) How do you describe the color of your teeth? → VAS: yellowish/grey (0.0 cm), white (10.0 cm)
  (10) How do you describe the brightness of your teeth? → VAS: dark (0.0 cm), bright (10.0 cm)
  (11) How satisfied are you with the color of your teeth? → VAS: very dissatisfied (0.0 cm), satisfied (10.0 cm).
  (12) How do you think other people assess your teeth/tooth color? → VAS: unattractive (0.0 cm), attractive (10.0 cm).
  Questions related to tooth sensitivity, tooth surface, and mouthfeel:
  (13) How sensitive are your teeth (cold beverages/food, ice, cold air, etc.)? → VAS: very high tooth sensitivity (0.0 cm), no tooth sensitivity (10.0 cm)
  (14) How do you assess the surface of your teeth (by using the tongue)? → VAS: rough (0.0 cm), smooth (10.0 cm).
  (15) How does your mouth feel? → VAS: unpleasant (0.0 cm), pleasant (10.0 cm)
  Comments:
  (16) Do you have further comments? (options: no, yes = free text)

### 2.5. Statistical Analysis

For each question, the median, mean, and standard deviations were calculated for all patients, using Microsoft Excel (Microsoft Excel, Version 1908, 2020). Statistical analyses were performed by using two-sided independent *t*-tests and calculation of 95% confidence intervals (CIs). Note that one patient did not answer questions 9–12 at follow-up (however, all other questions were answered and consequently this patient was included in the statistical analysis).

## 3. Results and Discussion

### 3.1. Patient Demographic Data and Oral Care/Tooth Whitening Habits (Questions 1–8)

25 patients met the inclusion criteria and were included into the study. Table 1 shows the demographic and general data of the patients. Most of the patients used electric toothbrushes and did not use special whitening products, for neither home nor in-office use.

### 3.2. Questions Related to Tooth Color (Questions 9–12)

Special questionnaires for analyzing the subjective efficacy of whitening products have been described [37]. In the present study, both medians and means improved after the 4-week use of the hydroxyapatite gel for the following parameters: “color of teeth”, “brightness of teeth”, and “satisfaction with tooth color” (Table 2); thus teeth seem to appear “healthier”. Interestingly, the subjective parameter “satisfaction with tooth color” was the whitening parameter which improved the most (Δ medians = 1.7 cm; Table 2; *p* = 0.1, 95% CI: (0.5; 1.7)). The brightness of the teeth increased as well (*p* = 0.9, 95% CI: (0.46; 1.29)). Taken together, this confirms results from in vitro studies that demonstrated tooth-whitening properties of calcium phosphates like hydroxyapatite [27,28,29], suggesting that hydroxyapatite is a promising whitening agent [6,29].

The whitening effect of biomimetic hydroxyapatite can be explained by:

(1) the formation of a white mineral (protective) layer on the enamel surface [24,26,29],
(2) the remineralization of enamel lesions (i.e., resulting in a smoother enamel surface) [7,8,9], and
(3) the reduction of plaque formation and exogenous stains (i.e., as a consequence, the incorporation of colorants into plaque and calculus is reduced) [1,4,13].

This demonstrates that biomimetic hydroxyapatite leads not only to an instant tooth-whitening effect but may also contribute to a long-lasting whitening effect (e.g., due to a smoother enamel surface and reduction of plaque formation after application of biomimetic hydroxyapatite). The mode of action of biomimetic hydroxyapatite regarding tooth-whitening can be explained by the formation of a white layer on the tooth surface. Consequently, teeth appear whiter and brighter after the application of hydroxyapatite [6].

Data from the literature indicate that this relationship is dose-dependent, i.e., both tooth brightness and tooth whiteness increase with rising hydroxyapatite content in the oral care formulation [31]. Unlike peroxide-based formulations, hydroxyapatite-based whitening formulations (e.g., toothpastes, mouthwashes, gels) can be easily applied by patients themselves by finger or toothbrush, have no side effects [18,19,22], and are relatively low-cost compared to in-office bleaching products/techniques. Currently, fluoride-free hydroxyapatite-based formulations do not have a regulated maximum daily dose, therefore they can be used as frequently as needed.

### 3.3. Questions Related to Tooth Sensitivity, Tooth Surface, and Mouthfeel (Questions 13–15)

Both medians and means for parameters “tooth sensitivity”, “tooth surface”, and “mouthfeel” improved after the 4-week use of the hydroxyapatite gel (Table 3). These results are in good agreement with a recent observational study analyzing a hydroxyapatite toothpaste [17]. Additionally, a recently published meta-analysis came to the conclusion that hydroxyapatite is the most efficient active ingredient to be used for prevention of dentin hypersensitivity [16]. Hydroxyapatite particles can occlude open dentin tubules [3,6,33], which leads to a clinical improvement of symptoms associated with dentin hypersensitivity [16,26,38,39]. Bleaching sensitivity is the most commonly reported side effect after using peroxide-based products [22,40,41]. In this study, dentin hypersensitivity was significantly reduced after the 4-week use of the hydroxyapatite oral care gel (*p* < 0.05, 95% CI: (0.8;2.4)). Additionally, the subjects reported smoother teeth after the use of the gel (*p* < 0.05, 95% CI: (0.54;1.6)).

There are two scientific explanations why patients reported smoother teeth. Firstly, hydroxyapatite remineralizes early enamel lesions homogeneously, which was shown in vitro and in situ [7,8,9]. Secondly, hydroxyapatite reduces bacterial attachment to the enamel surface, i.e., plaque formation is reduced [1,4,15].

### 3.4. Further Comments of the Patients (Question 16)

16 patients did not have any further comments, 8 patients had some remarks, and 1 patient did not answer this question. Note that 1 patient had four comments (Table 4).

## 4. Conclusions

This 4-week observational pilot study demonstrated the general suitability of this hydroxyapatite-based oral care gel to act as an adjunct to daily tooth brushing. It has the potential to be used for daily tooth whitening at home. However, besides this observational study analyzing subjective parameters, future studies should analyze if this hydroxyapatite gel could positively influence quality of life as well as compliance for daily oral care. Due to the character of this pilot study (e.g., a small number of patients, no control group(s), no blinding) and the large variability, future clinical trials should be aimed at analyzing the whitening effects of hydroxyapatite-based products using standardized objective techniques (e.g., visual assessment using shade guides or analysis of digital images) and to use suitable control groups (e.g., peroxide-based formulations). It is important to note that each individual has his/her “personal” tooth color. There are different shades of white/yellow for each person. Consequently, this tooth color impacts the outcome of a whitening perception. Naturally brighter teeth will be brighter after the use of whitening products. In contrast to this, naturally yellow teeth will always display a more yellowish color, even after the use of (in office/at home) whitening products.

Unlike “traditional” whitening agents (e.g., peroxides, toothpaste abrasives with a high hardness, and blue covarine) [42], particulate hydroxyapatite has an excellent biocompatibility, is not harmful to enamel or exposed dentin, and consequently can be used daily [6,18,19,20,21]. In addition to its whitening properties [29], biomimetic hydroxyapatite provides other benefits for preventive oral health care, like enamel remineralization [7,8,9], reduction of bacterial colonization of tooth surfaces [1,13], and protection from dentin hypersensitivity [3,17,38,43].

## Figures and Tables

**Table 1:** Patient demographic data and oral care/tooth whitening habits.

1. Age	Median: 47 years Mean: 46 ± 16 years
2. Total number of subjects (number of males/females)	25 (5/20)
3. Toothpaste used during the study	- 6 Biorepair (Dr. Kurt Wolff GmbH & Co. KG, Bielefeld, Germany) (main active ingredient: hydroxyapatite) - 5 Aronal/Elmex/Meridol (CP GABA GmbH, Hamburg, Germany) (main active ingredient: fluoride) - 5 Oral-B (Procter & Gamble, Schwalbach, Germany) (main active ingredient: fluoride) - 4 Colgate (CP GABA GmbH, Hamburg, Germany) (main active ingredient: fluoride)- 2 Odol med 3 (GlaxoSmithKline Consumer Healthcare GmbH & Co. KG, Munich, Germany) (main active ingredient: fluoride) - 1 Signal (Unilever Deutschland GmbH, Hamburg, Germany) (main active ingredient: fluoride) - 1 Prokudent (Dirk Rossmann GmbH, Burgwedel, Germany) (main active ingredient: fluoride) - 1 Unknown
4. Electric toothbrush/manual toothbrush/sonic/ultrasonic toothbrush	19 electric toothbrush, 6 manual toothbrush, 2 sonic/ultrasonic toothbrush (note that two patients used both electric and manual toothbrush)
5. Whitening product	22 no whitening product, 3 whitening toothpaste, 0 whitening mouthwash, 0 whitening stripes, 0 other whitening products
6. Frequency of usage of whitening products	22 never, 2 less than once a month, 1 1–3× per month, 0 1–3× per week, 0 4–6× per week, 0 daily
7. Frequency of usage of in-office bleaching products	25 never, 0 1× per year, 0 2× per year, 0 more than 2× per year
8. Unwanted effects of bleaching	18 no bleaching was performed, 1 I do not feel any unwanted effects, 6 sensitive teeth, 1 gum problems, 1 unknown (note that two patients marked two answers)

**Table 2:** Subjective parameters related to tooth color; baseline and follow-up (4 weeks) data (VAS = visual analogue scale).

Questions	Baseline VAS in cm	Follow-Up VAS in cm	Δ Medians in cm	p-Value
▪9. Color of teeth yellowish/grey (0.0 cm) … white (10.0 cm)	Median: 5.8	Median: 6.3	+ 0.5	0.14
	Mean: 5.5 ± 2.3	Mean: 6.4 ± 1.9		
▪10. Brightness of teeth dark (0.0 cm) … bright (10.0 cm)	Median: 6.3	Median: 6.9	+ 0.6	0.09
	Mean: 5.9 ± 1.9	Mean: 6.8 ± 1.7		
▪11. Satisfaction with tooth color very dissatisfied (0.0 cm) … satisfied (10.0 cm)	Median: 5.1	Median: 6.8	+ 1.7	0.1
	Mean: 5.4 ± 2.7	Mean: 6.5 ± 2.0		
▪12. Assessment of tooth color by other persons unattractive (0.0 cm) … attractive (10.0 cm)	Median: 6.9	Median: 6.4	- 0.5	0.6
	Mean: 6.3 ± 2.3	Mean: 6.6 ± 1.9		

**Table 3:** Subjective parameters related to tooth sensitivity, tooth surface, and overall mouthfeel; baseline and follow-up (4-week) data (VAS = visual analogue scale).

Questions	Baseline VAS in cm	Follow-Up VAS in cm	Δ Medians in cm	*p*-Value
▪13. Tooth sensitivity very high tooth sensitivity (0.0 cm) … no tooth sensitivity (10.0 cm)	Median: 5.1	Median: 7.5	+ 2.4	0.04
	Mean: 5.3 ± 2.9	Mean: 6.9 ± 2.7		
▪14. Tooth surface rough (0.0 cm) … smooth (10.0 cm)	Median: 7.6	Median: 8.4	+ 0.8	0.008
	Mean: 7.4 ± 1.6	Mean: 8.5 ± 1.0		
▪15. Overall mouthfeel unpleasant (0.0 cm) … pleasant (10.0 cm)	Median: 7.5	Median: 8.2	+ 0.7	0.19
	Mean: 7.2 ± 1.9	Mean: 7.9 ± 1.8		

**Table 4:** Comments of the patients after follow-up (4-week; clustered from free text answers).

Comments	Number of Patients
No comments	16
General problems in applying the gel	3
No changes in subjective parameters	2
Pleasant mouthfeel	1
Pleasant taste	1
Pleasant mouthfeel (in combination with a hydroxyapatite toothpaste)	1
No pain when eating hot/cold meals	1
No staining (coffee, tea)	1
Application by a soft toothbrush (instead of finger)	1
No answer to this question	1
